# Aminosilane Functionalization and Cytotoxicity Effects of Upconversion Nanoparticles Y_2_O_3_ and Gd_2_O_3_ Co-Doped with Yb^3+^and Er^3+^

**DOI:** 10.5772/62252

**Published:** 2016-01-01

**Authors:** Dalia Holanda Chavez, Karla Juarez-Moreno, Gustavo Alonso Hirata

**Affiliations:** 1 Center of Scientific Research and Higher Education of Ensenada, BC (CICESE), México; 2 Center of Nanoscience and Nanotechnology (CNyN), México; 3 CONACYT Research Fellow at Center of Scientific Research and Higher Education of Ensenada, BC, Mexico

**Keywords:** Upconversion, Nanoparticles, Sol-gel, Biolabels, Aminosilane Functionalization

## Abstract

In this study, luminescent upconversion nanoparticles (UCNPs) Y_2_O_3_ and Gd_2_O_3_ co-doped with Yb^3+^ and Er^3+^ were prepared by the sol-gel method (SG). These NPs are able to absorb near infrared photons and upconvert them into visible radiation with a direct application in bioimaging, as an important tool to diagnose and visualize cancer cells. The UCNPs were coated with a thin silica shell and functionalized with amino groups for further folic acid conjugation to allow their interaction with folate ligands on the cell surface. Their physical properties were analysed by Transmission Electron Microscopy (TEM), Fourier transform infrared spectroscopy (FTIR) and photoluminescence (PL) measurements. The PL results revealed excellent luminescence properties on all core-shell UCNPs. Cytotoxicity experiments with concentrations of bare and aminosilane coated/functionalized UCNPs between 0.001 μg/mL to 1 μg/mL were tested on two different cell lines from human cervix carcinoma (HeLa) and human colorectal adenocarcinoma (DLD-1) with a colorimetric assay based on the reduction of MTT reagent (methy-134-thiazolyltetrazolium). The assays show that some concentrations of bare UCNPs were cytotoxic for cervical adenocarcinoma cells (HeLa); however, for human colorectal adenocarcinoma all UCNPs are non-cytotoxic. After UCNPs functionalization with silica-aminosilane (APTES/TEOS), all of the nanoparticles tested were found to be non-cytotoxic for both cell lines. The UCNPs functionalized in this work can be further conjugated with specific ligands and used as biolabels for detection of cancer cells.

## 1. Introduction

Luminescence upconversion nanoparticles (UCNPs) are usually fabricated by combining two rare earth ions in a proper host; the first ion (acting as a sensitizer) absorbs near infrared (NIR) radiation and transfers the energy to the second one; as a result, the nanoparticle emits the excess energy as photons in the visible range. This phenomenon is called energy transfer upconversion (ETU) [1]. The UCNPs have a lanthanide base and numerous studies use them as luminescent biolabels or as contrast agents in magnetic resonance imaging (MRI). As a comparison with conventional fluorescent materials, such as organic dyes or fluorescent proteins, the advantage of the UCNPs is that they do not fade, so they can be used for longer [2–4]. Furthermore, some of the fluorescent materials are generally excited with ultraviolet light, which may cause cell death or DNA damage [5]. The UCNPs with the hosts Y_2_O_3_ and Gd_2_O_3_ co-doped with Er^3+^/ Yb^3+^ have been prepared by sol-gel synthesis (SG) [6, 7]. The ion Yb^3+^ absorbs NIR radiation and transfers a first photon to Er^3+^ and the electron is promoted to ^4^I_11/2_ level from ^4^I_15/2_ level. A second photon is transferred to Er^3+^ to raise the electron to a higher level ^4^S_3/2_. When it decays to its base state it emits green light. The electron can also be promoted to level ^4^F_9/2_, if the percentage of the doping of Yb^3+^ changes and the emission is in red [8]. The UCNPs were coated with a thin silica core-shell by Stöber method [9] and functionalized with amine group (APTES/TEOS) to enable folic acid conjugation [10]. We used a combination of two methods: the first was to coat the UCNPs with a thin silica shell; the use of a surfactant was needed in order to avoid agglomeration of the nanoparticles during the process. The second method was to perform the aminosilane functionalization of the UCNPs. The luminescent properties were studied; their morphology was also analysed by transmission electron microscopy (TEM) and Fourier transform infrared spectroscopy (FTIR) to confirm amine functionalization. Cytotoxicity assay was performed in a 96-well plate containing 10,000 cells per well. Viability of the two cancer cell lines was analysed by a colorimetric assay based on the MTT reagent (methy-134 thiazolyltetrazolium), after 24 hr post incubation with the UCNPs [4]. The silica-coated UCNPs obtained had strong luminescence and size; they were also non-cytotoxic, allowing them to be further used as biolabels in cancer cell lines such as human cervix carcinoma (HeLa) with the appropriate folic acid functionalization.

## 2. Materials and methods

The UCNPs of Gd_2_O_3:_Er^3+^/Yb^3+^ and Y_2_O_3:_Er^3+^/Yb^3+^ were prepared by the sol-gel method (SG) with concentrations of Yb^3+^ (1% and 10% mol) and Er^3+^ (1% mol) and annealing temperatures of 900°C for Gd_2_O_3_ and 1,200°C for Y_2_O_3_. The description of the methodology for both hosts is in our previous article; these UCNPs were selected due to their improved luminescence compared with others prepared [6]. The UCNPs were ultrasonicated with 20 ml of isopropanol/ethanol with the ultrasonic liquid processor (Sonics & Materials, Inc.) at 70% of the amplitude for about 30 minutes before the analysis for TEM or silica coating, to avoid agglomeration of the nanoparticles. They were characterized by X-Ray diffraction (XRD) by the diffractometer Phillips X'Pert-MPD [6]. The morphology and the nanoparticle size for the silica-coated UCNPs were also studied. Their photoluminescence was analysed with a spectrofluorometer. Silica-coating and aminosilane functionalization was done by APTES/TEOS technique [10].

### 2.1 Silica-coating and aminosilane functionalization

Surface silanization is an important method for the UCNPs surface modification. The modified silanes, such as aminopropyltrimethoxysilane (APTES), can make the UCNPs water soluble and able to be conjugated with biological molecules. Wilhelm and associates prepared UCNPs coated with a silica shell and protein conjugation [11–12]; Li also prepared UCNPs with a thin silica shell [13]. The silanized UCNPs are more stable in solutions than polymeric coats and the risk of cytotoxic effect is also reduced [5]. The purpose of the aminosilane functionalization is to conjugate the UCNPs with biological moieties that recognize cell surface receptors. It is important that the ligand of the UCNPs has high affinity and specificity, to enable it to bind to the cell and penetrate into the membrane to identify it. The amino groups enable folic acid (FA) conjugation. Some cancer cells, such as cervical adenocarcinoma (HeLa) have overexpression of FA on the cell surface [2].

Silica coating of the nanoparticles was done by Stöber synthesis [8]. Distilled water (100 ml) was mixed with 10 mmol concentration of the UCNPs under constant magnetic stirring for 20 min. Separately, 30 ml of ethanol was mixed and agitated for 20 min with 0.6 ml of TEOS (Tetraethyl orthosilicate, Sigma Aldrich 98%). Another solution containing 1 mol ammonium hydroxide (NH_4_OH, Sigma Aldrich), 0.2 ml of surfactant IGEPAL (Sigma Aldrich) and distilled water was stirred; this surfactant was needed in order to minimize the UCNPs agglomeration during the coating process. The solutions were mixed together and agitated for 24 hr. The silica coated UCNPs were precipitated with acetone and centrifuged three times. Finally, the UCNPs were filtered and then annealed at 900°C for 2 hr. The UCNPs were ultrasonicated again for 15 min. The aminosilane functionalization process was done by mixing the silica-coated UCNPs with ethanol, 0.02 ml of 3-Aminopropyl- trimethoxysilane (APTES, 98% Sigma Aldrich), 0.14 ml of TEOS and 0.2 ml of ammonium hydroxide for approximately 4 hours at 24°C [10]. The functionalized UCNPs were dried and collected. FTIR analysis was performed to ascertain that the amine functionalization was correctly carried out and TEM analysis was done to verify the core-shell on the UCNPs.

### 2.2 Characterization

The crystallinity of the UCNPs was analysed by XRD with the Phillips X'Pert-MPD, equipped with Cu Kα radiation (λ= 0.15406 nm). Measurements scanned over 2θ range of 10–80° were taken with a step size of 0.1° and a 1 s dwell per point. The results obtained were compared with the database PCPDFWIN [6]. The transmission electron microscopy (TEM) used was JEOL JEM-2100-F, to study the morphology, the nanoparticle size and the presence of the silica-coating. The photoluminescence of the UCNPs was analysed with a fluorescence spectrometer (PL, Hitachi® FL-4500) with 980 nm for excitation. FTIR analysis was done to confirm amine functionalization (Thermonicolet 1700).

### 2.3 Cell culture

Human cervix carcinoma HeLa cells (ATCC CCL-2) and human colorectal adenocarcinoma DLD-1 cells (ATCC CCL-221) were obtained from the American Type Culture Collection (ATCC). The cells were cultivated in RPMI-1640 media supplemented with 10% Fetal Bovine Serum (FBS, BenchMark, Gemini Bio Products), 1% Penicillin streptomycin (Sigma-Aldrich), 1% L-glutamine and 1.5 g/l sodium bicarbonate. The cells were propagated in growth medium and maintained at 37°C and 5% CO_2_.

### 2.4 Cytotoxicity assay

Cytotoxicity is used to test whether reagent affects the viability of a cell and causes cellular damage and cell death. Several authors have developed *in vitro* tests to predict the toxic effects of some drugs and chemical compounds, including nanoparticles [14]. To evaluate the potential of silica coating UCNPs for biomedical applications, we compared the cytotoxic activity of bare and coated UCNPs on two different cancer cell lines. Viability of HeLa and DLD-1 cell lines was analysed by a colorimetric assay based on the reduction of the MTT reagent (methy-134 thiazolyltetrazolium) by using the TOX1 in vitro toxicology assay kit (Sigma-Aldrich). The cytotoxicity test was performed in a 96-well plate containing 10,000 cells per well. The UCNPs were ultrasonicated and diluted at 0.001 μg/mL to 1 μg/mL in RPMI-1640 media. The cells were incubated with different amounts of UCNPs for 24 hr at 37°C and 5% CO_2_. The incubation of the cells in complete RPMI-1640 media without UCNPs acted as a positive control, simulating cell behaviour under ideal conditions. DMSO (Dimethyl sulphoxide) was used to induce cell death. After the incubation time, the cells were washed with phosphate buffer solution pH 7.4 (PBS 1x) and MTT reagent was added to the plate following the instructions of the manufacturer. Cytotoxicity was assessed by absorbance measurements with an ELISA plate reader (Thermo Scientific, USA) at 570 and 690 nm. All data obtained from incubated UCNPs were normalized to data from three positive control wells with no Gd_2_O_3_:Er^3+^/Yb^3+^ and no Y_2_O_3_:Er^3+^/Yb^3+^ in each independent experiment [4].

### 2.5 Statistical analysis

All experiments were done in triplicate. The results were expressed as mean ± standard deviation of three independent experiments. Data were evaluated by analysis of variance (ANOVA), followed by Tukey's Multiple Comparison Test, using Graph Pad Prism 6.0 software. The results were considered statistically significant when p < 0.05.

## 3. Results

This section shows crystallinity of the UCNPs by XRD and the morphology analysis performed by TEM, the photoluminescence of all UCNPs synthesized in this work and the FTIR analysis. The cytotoxicity effect of the UCNPs was also analysed in human cervix carcinoma (HeLa) and human colorectal adenocarcinoma (DLD-1) with colorimetric assay based on the reduction of the MTT reagent (methy-134-thiazolyltetrazolium).

### 3.1 XRD and morphology of Y2O3:Er3+/Yb3+ and Gd2O3:Er3+/Yb3+ UCN

**Figure 1.**
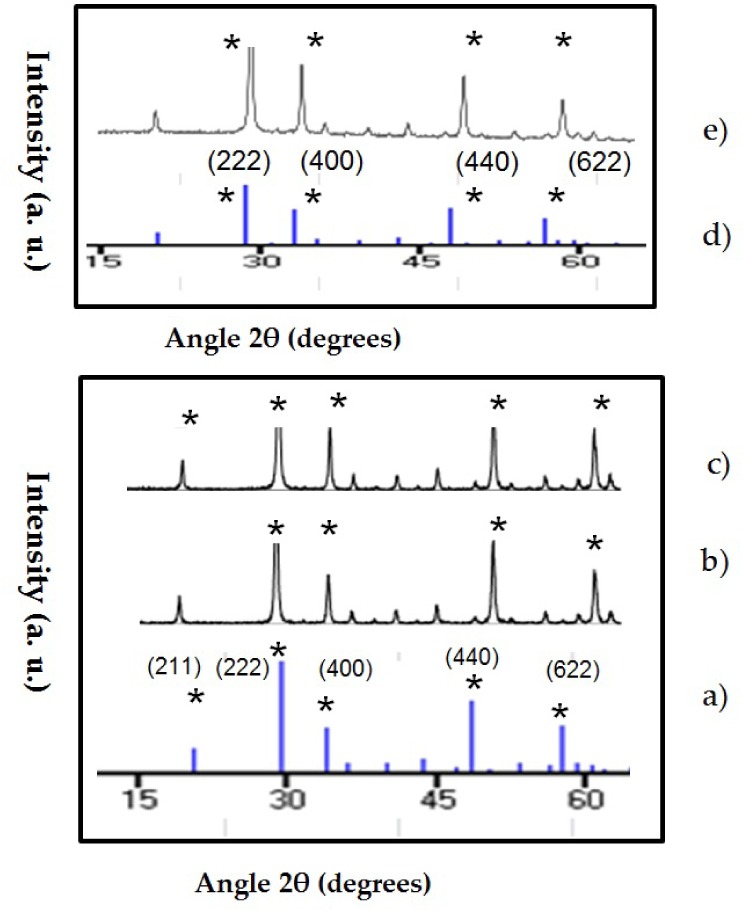
XRD patterns of Y_2_O_3_: Er^3+^/Yb^3+^ and Gd_2_O_3_: Er^3+^/Yb^3+^ compared with the standard data JCPDS no. 89-5592 [Y_2_O_3_] and JCPDS no. 88-2165 [Gd_2_O_3_]. a) JCPDS no. 89-5592 [Y_2_O_3_] cubic structure, b) Y_2_O_3_:Er^3+^/Yb^3+^ (1%, 1%), c) Y_2_O_3_: Er^3+^/ Yb^3+^ (1%, 10%), d) JCPDS 88-2165:Gd_2_O_3_ cubic structure, e) G_2_O_3_: Er^3+^/Yb^3+^ (1%, 10%). The peaks match in intensity to the JCPDS data; all the samples have cubic structure.

The composition obtained for the UCNPs synthesized by sol-gel method was (Gd_1-x-__y_Yb_x_Er_y_)_2_O_3_ and _(_Y_1-x-__y_Yb_x_Er_y_)_2_O_3_ (Table_1). The XRD patterns of the UCNPs are in Figure 1. All the diffraction peaks are consistent with the JCPDS No. 89-5592 (Y_2_O_3_) and JCPDS No. 88-2165 for Gd_2_O_3_ database and demonstrate that the samples have a cubic structure.

**Table 1.** Doping and annealing temperatures for the UCNPs of Gd_2_O_3_ and Y_2_O_3_

Number	Host	% Er^3+^	% Yb ^3+^	Temperatures for annealing (°C)	Time of annealing (hr)
		*y*	*x*		
1	Y_2_O_3_	1	1	1,200	2

2	Y_2_O_3_	1	10	1,200	2

3	Gd_2_O_3_	1	10	900	2

**Figure 2.**
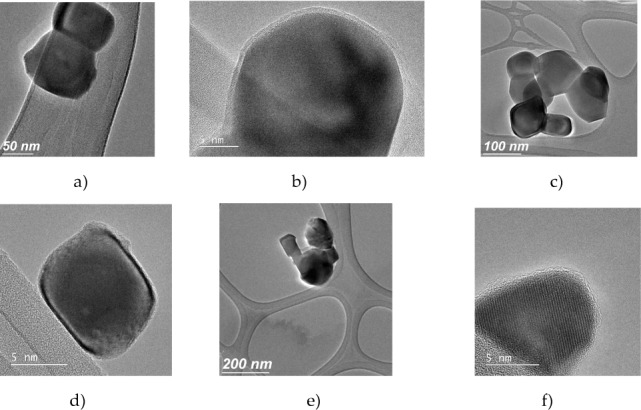
TEM images of (a) bare Y_2_O_3_: Er^3+^,Yb^3+^ (1%, 1%); (b) aminosilane functionalization showing the fine silica coating of Er^3+^ 1%, Yb^3+^ 1%; (c) bare Er^3+^ 1%, Yb^3+^ 10%; (d) aminosilane Er^3+^ 1%, Yb^3+^ 10%; (e) bare Gd_2_O_3_: Er^3+^, Yb^3+^ and (f) Gd_2_O_3_: Er^3+^ 1%, Yb^3+^ 10% with aminosilane functionalization.

Figure 2 represents the TEM images of bare (2_a, 2_c and 2_e) and aminosilane UCNPs (2_b, 2_d and 2_e). The size increased only by about 5 nm due to the core-shell formed of silica. Some of the UCNPs presented a spherical shape. In Figure 2_b and 2_f the crystallographic planes are clearly shown and the silica coating can be seen as amorphous. The UCNPs tend to agglomerate after and under coating. The Gd_2_O_3_ UCNPs presented an average size of 50 nm and the Y_2_O_3_ have an average size of 70 nm.

### 3.2 Upconversion luminescence

**Figure 3.**
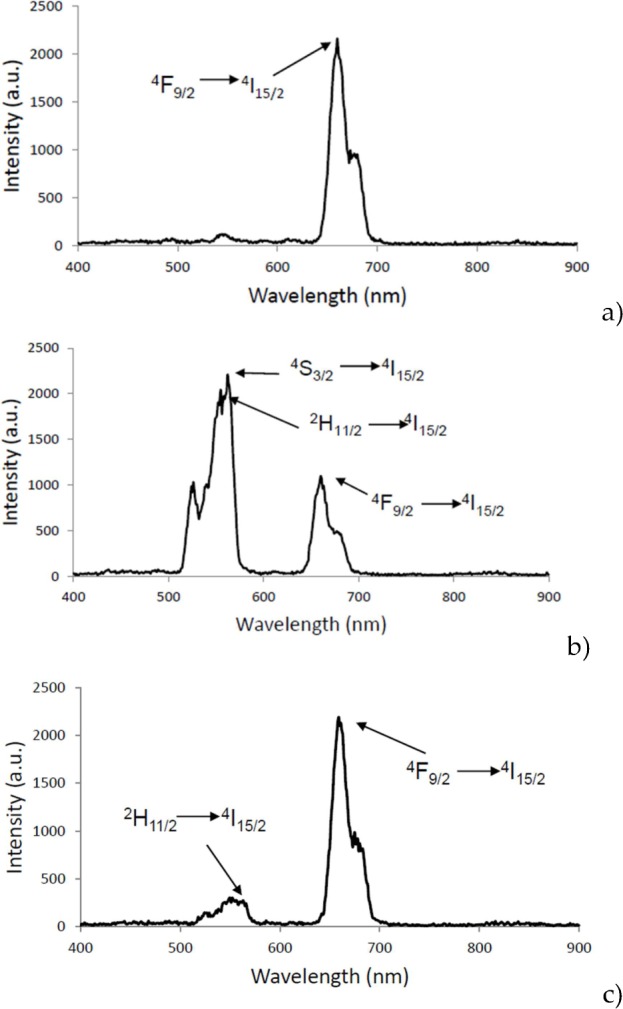
The upconversion emission spectra, under 980 nm excitation for (a) Gd_2_O_3_:Er^3+^/ Yb^3+^ (1%, 10%), red emission (660–662 nm), for (b) Y_2_O_3_:Er^3+^/Yb^3+^ (1%, 1%) green emission (564 nm) in ^4^S_3/2_→^4^I _15/2_ transition and (c) Y_2_O_3_:Er^3+^/ Yb^3+^ (1%, 10%) red emission (661 nm) in ^4^F_9/2_→^4^I_15/2_ transition

Upconversion luminescence spectra of aminosilane UCNPs, under 980 nm laser excitation, are shown in Figure 3 for all doping concentrations. For Gd_2_O_3_:Er^3^+, Yb^3^+ (Figure 3_a), the spectra show a peak at 662 nm (red emission), which is attributed to transition ^4^F_9/2_ → ^4^I_15/2_ of Er^3+^ ion. For Y_2_O_3_:Er^3+^, Yb^3+^ UCNPs, transitions ^2^H _11/2_ → ^4^I_15/2_ (550 nm), ^4^S_3/2_ → ^4^I_15/2_ (564 nm) and ^4^F_9/2_→^4^I_15/2_ (660 nm) are present (Figure 3_b and 3_c). Green emission (564 nm) is present in the co-doping Er^3+^ (1%) and Yb^3+^ (1%). For the Er^3+^ (1%) and Yb^3+^ (10%) co-doping, the highest emission is in red (663 nm) and corresponds to the transition ^4^F_9/2_→^4^I_15/2_; the green transitions are present but with lower intensity.

### 3.3 FTIR analysis

**Figure 4.**
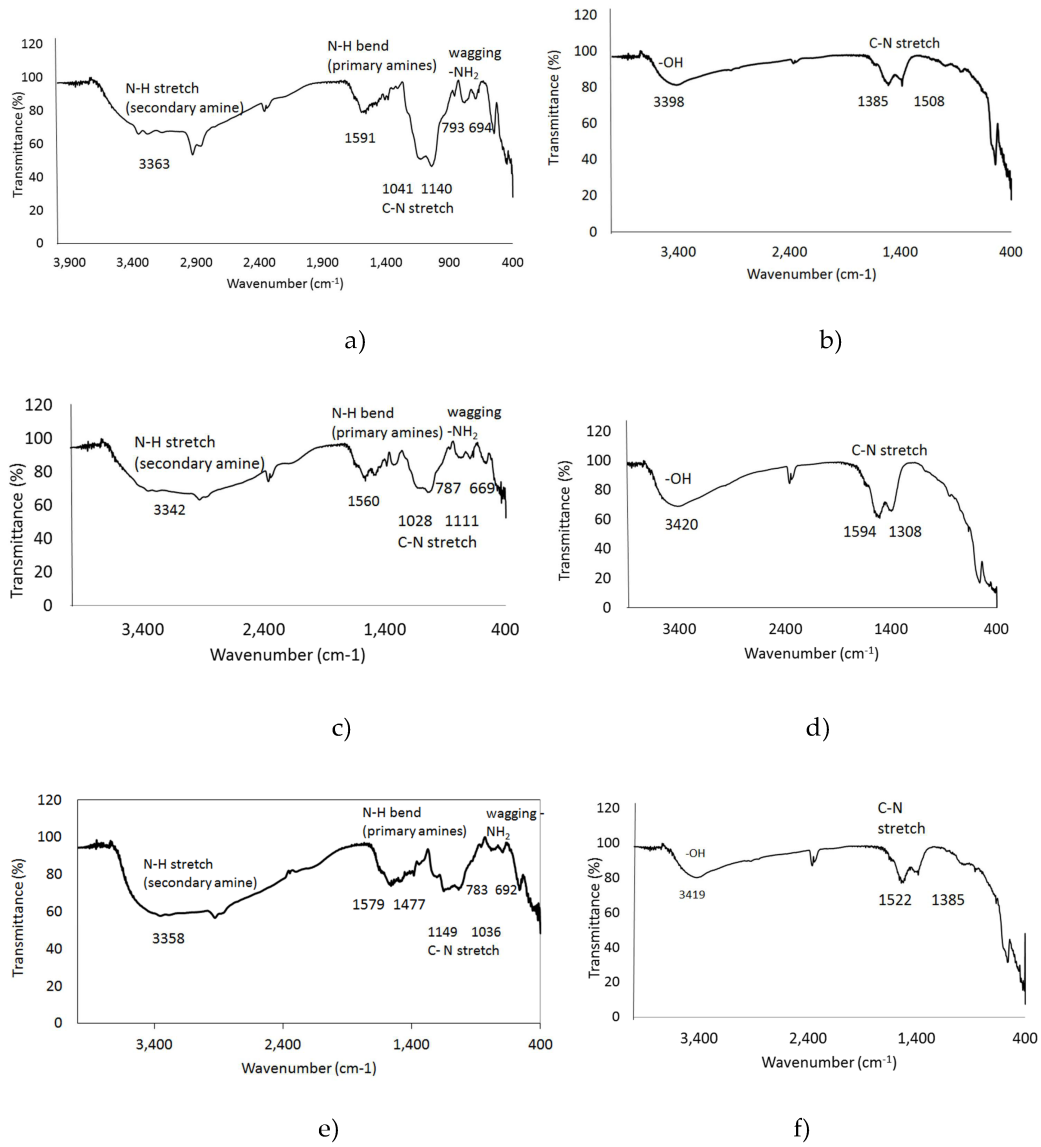
FTIR spectra of aminosilane functionalization UCNPs and bare UCNPs of a) Gd_2_O_3_:Er^3+^/Yb^3+^ (1%, 10%) functionalized, b) bare Gd_2_O_3_:Er^3+^/Yb^3+^ (1%, 10%), c) Y_2_O_3_:Er^3+^/Yb^3+^ (1%, 1%) functionalized, d) bare Y_2_O_3_:Er^3+^/Yb^3+^ (1%, 1%), e) Y_2_O_3_:Er^3+^/Yb^3+^ (1%, 10%) functionalized and f) bare Y_2_O_3_:Er^3+^/Yb^3+^ (1%, 10%). The presence of the amino groups is shown in the N-H stretch of the secondary and primary amines.

The Fourier transform infrared spectroscopy analysis (FTIR) was used to verify the functionalization achieved with the APTES/TEOS method, to confirm the presence of the amine groups on the UCNPs. The amine groups can be further used to bind folic acid (FA). It is well known that HeLa and other cancer cells overexpress the receptor for FA; therefore, UCNPs functionalized with FA will be attached to its receptor, the cells will be able to internalize them into the cytoplasm and it would be possible to identify the cells by the luminescence The presence of the amino groups on the surface of the UCNPs for Gd_2_O_3_:Er^3+^/Yb^3+^ (1%, 10%), Y_2_O_3_:Er^3+^/Yb^3+^ (1%, 1%) and Y_2_O_3_:Er^3+^/Yb^3+^ (1%, 10%) is shown in Figure 4a,4c and 4e respectively. For comparison purposes, the FTIR analysis for bare UCNPs was also done, as depicted in Figure 4b,4d and 4f.

### 3.4 Cytotoxic assay

**Figure 5.**
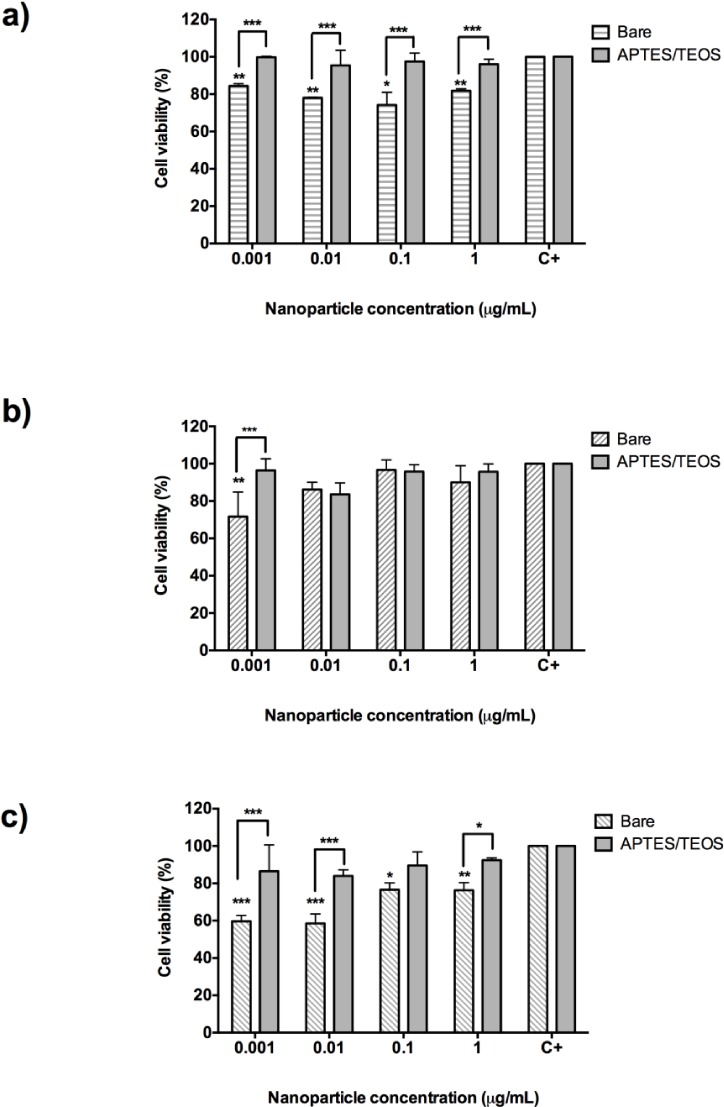
Cell viability assay with 24-hour incubation of bare (pattern bars) and aminosilane (grey bars) UCNPs with HeLa cells. a) Gd_2_O_3_:Er^3+^/Yb^3+^ (1%, 10%), b) Y_2_O_3_:Er^3+^/Yb^3+^ (1%, 1%) and c) Y_2_O_3_:Er^3+^/Yb^3+^ (1%, 10%). Results represent the mean and standard deviation of a threefold independent experiment. *p<0.05; ** p<0.01 and ***p<0.001.

The graphics in Figure 5 show the cytotoxicity results for bare and aminosilane UCNPs incubated on HeLa cells. As shown, bare Gd_2_O_3_ UCNPs reduced cell viability by 20%; however, APTES/TEOS-functionalized UCNPs do not cause any cytotoxic effect on HeLa cells (p=0.0071) (Figure 5_a). The smooth cytotoxic effect of 30% and 40% cell death caused by bare Gd_2_O_3_ UCNPs at 0.01 μg/mL (Figure 5_a) and at 0.001 μg/mL by Y_2_O_3_ (Figure 5_b and 5_c) respectively may be explained by susceptibility to the manipulation during the experiment. In the case of Y_2_O_3_:Er^3+^/Yb^3+^ (1%, 1%) and Y_2_O_3_:Er^3+^/Yb^3+^ (1%, 10%), it was observed that bare nanoparticles tend to agglomerate and precipitate after the addition to the well of the 96-well plate. Even after they have been ultrasonicated, this effect may contribute to damaging the cells by mechanical forces. In some cases, lower concentrations of UCNPs (0.001 and 0.01 μg/mL) showed less cell viability than bigger concentrations. This behaviour does not reflect an increase in cell viability, but an agglomeration of nanoparticles in the bottom of the plate that may interfere with the absorbance reading of the plate. However, as shown in Figure 5_b and 5_c, none of the functionalized UCNPs have cytotoxic effects on HeLa cells. Remarkably, both Gd_2_O_3_ and Y_2_O_3_:Er^3+^/Yb^3+^(1%, 10%) nanoparticles functionalized with APTES/TEOS (grey columns) have no cytotoxic effect on HeLa cells.

**Figure 6.**
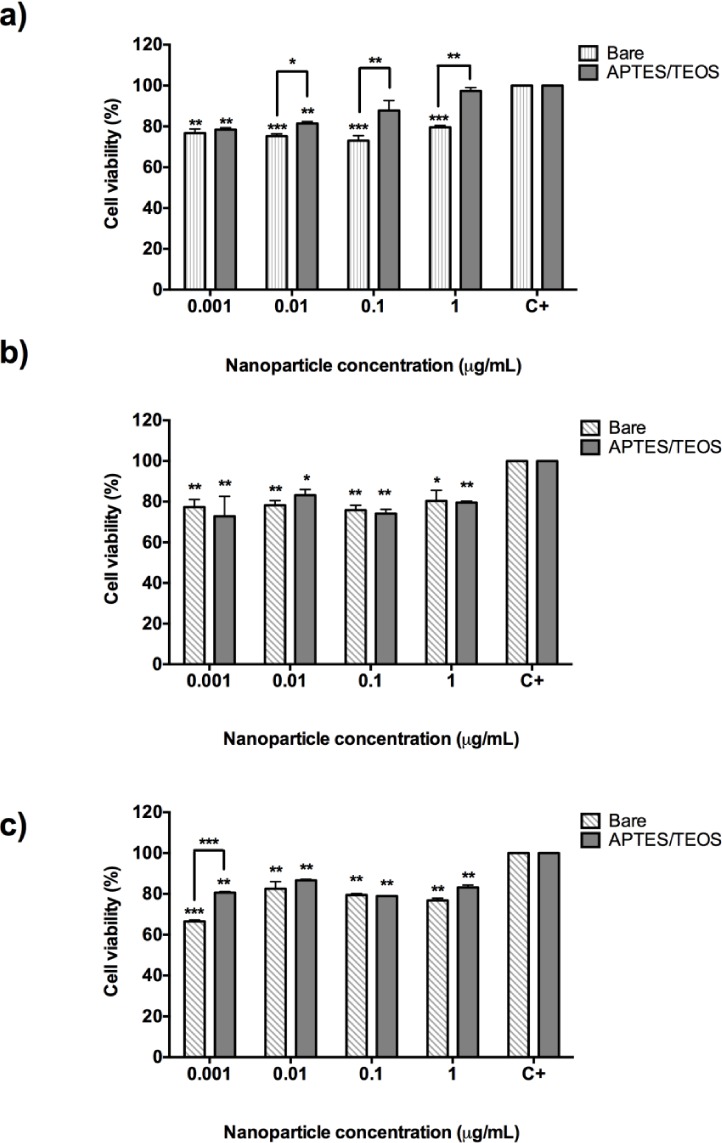
MTT assay with 24-hour incubation of bare and aminosilane UCNPs with colorectal adenocarcinoma (DLD-1) cells. a) Gd_2_O_3_:Er^3+^/Yb^3+^ (1%, 10%), b) Y_2_O_3_:Er^3+^/Yb^3+^ (1%, 1%) and c) Y_2_O_3_:Er^3+^/Yb^3+^ (1%, 10%). *p<0.05; ** p<0.01 and ***p<0.001.

Figure 6 shows the cytotoxicity results for bare and aminosilane UCNPs incubated with human colorectal adenocarcinoma (DLD-1) cells. Incubation of bare Gd_2_O_3_:Er^3+^/Yb^3+^ (1%, 10%) nanoparticles with DLD-1 cells resulted in causing approximately 80% of cell survival (p=0.008) at concentrations from 0.001 μg/mL to 1 μg/mL. However, when UCNPs were functionalized with APTES/TEOS, it resulted in a reduction of the cytotoxic effect, as shown in Figure 6_a, where HeLa cells' survival increases almost to the values of untreated cells, which reflects an absence of cytotoxicity.

In the case of bare Y_2_O_3_:Er^3+^/Yb^3+^ (1%, 1%) nanoparticles (Figure 6_b) and bare Y_2_O_3_:Er^3+^/Yb^3+^ (1%, 10%), this causes a cell survival rate of 80% in the majority of the concentrations tested. DLD-1 cells proved to be slightly sensitive to concentrations of 0.001 to 1 μg/mL which causes a cell death rate of 25% (Figure 6_b and 6_c). However, after APTES/TEOS functionalization, the incubation of DLD-1 cells with the same concentration resulted in slightly reducing the cell death at higher concentrations of 1 μg/mL (20% cell death). These results corroborate the fact that, after APTES/TEOS functionalization, the nanoparticles proved to be non-cytotoxic and can be further attached to specific ligands for cell delivery to other types of cancer cells.

## 4. Discussion and conclusions

The UCNPs were prepared by the sol-gel method. The nanoparticles are mostly spherical in shape; the size of 50 nm was obtained for Gd_2_O_3_ and 70 nm for Y_2_O_3_. Surface modification by silica-coating was approximately 5 nm thick on all the UCNPs, being a suitable tool to improve the chemical durability of oxides. The nanoparticles tend to agglomerate after and during coating; a surfactant was added to have a homogeneous coating on all UCNPs. The aminosilane UCNPs showed good emission spectra in comparison with bare nanoparticles. Red emission was obtained on the UCNPs Gd_2_O_3_:Er^3+^/Yb^3+^ (1%, 10% mol) and also on the Y_2_O_3_:Er^3+^/Yb^3+^ (1%, 10% mol). On the other hand, the UCNPs Y_2_O_3_:Er^3+^/Yb^3+^ (1%, 1% mol) yielded high-intensity green luminescence. The peaks can be attributed to the transitions ^2^H _11/2_→ ^4^I _15/2_ (525–550 nm), ^4^S_3/2_→^4^I _15/2_ (564 nm) and ^4^F_9/2_→^4^I_15/2_ (660–670 nm). FTIR spectra showed the presence of the amino groups on the surface of the UCNPs, such as the N-H stretch of the secondary amines (3340–3363 cm^−1^), the primary amines (1571–1591 cm-^1^) and the wagging –NH_2_ (669–793 cm^−1^).

Referring to cytotoxicity assay, MTT reagent is taken up by cells and reduced by the enzyme succinic mitochondrial dehydrogenase to its insoluble form formazan, which is violet in colour and can be measured by spectroscopy. The crystals of formazan are retained in biochemically active cells and can be released by solubilizing them with dimethyl sulphoxide. In this way, the amount of MTT reduced by the living cells is quantified by a colourimetric method; thus, the ability of cells to reduce MTT is an indicator of mitochondrial integrity and its functional activity is interpreted as measuring cell viability. Determining the ability of cells to reduce MTT to formazan after exposure to a compound provides information about the toxicity of the UCNPs evaluated [14].

Cytotoxicity assays based on MTT revealed that HeLa cells' incubation with different amounts of bare Gd_2_O_3_:Er^3+^/Yb^3+^ (1%, 10%) and Y_2_O_3_:Er^3+^/Yb^3+^ (1%, 10%) nanoparticles proved to be non-cytotoxic, while incubation with bare Y_2_O_3_:Er^3+^/Yb^3+^ (1%, 1%) at 0.001 μg/mL caused a cell death rate of 40%. However, after the nanoparticles were functionalized with APTES/TEOS, all aminosilane UCNPs had more than 80% cell viability on HeLa cells; thus the cell death and the cytotoxic effect was reduced for all of the tested concentrations (0.001 μg/mL to 1 μg/mL).

For colorectal adenocarcinoma cells (DLD-1), incubation with bare UCNPs Y_2_O_3_:Er^3+^/Yb^3+^ (1%, 1%), Y_2_O_3_:Er^3+^/Yb^3+^ (1%, 10%) and Gd_2_O_3_:Er^3+^/Yb^3+^ (1%, 10%) showed that none of the tested concentrations resulted in a cytotoxic effect. Therefore, after functionalization with aminosilane, all the nanoparticles reduced their toxic effect at all concentrations (0.001 μg/mL to 1 μg/mL). In this sense we have proved that UCNPs' functionalization with aminosilane reduced their cytotoxic effect. Therefore, with the appropriate folic acid conjugation with the amino groups, these nanoparticles can be used as a biolabels to identify HeLa and other breast cancer cells. Further investigation is underway to establish a valid conclusion and a proof of principle of the cell interaction with the UCNPs nanoparticles.

## 6. Conflicts of interest

The authors declare no conflicts of interest.
